# Unmasking viral sequences by metagenomic next-generation sequencing in adult human blood samples during steroid-refractory/dependent graft-versus-host disease

**DOI:** 10.1186/s40168-020-00953-3

**Published:** 2021-01-24

**Authors:** M. C. Zanella, S. Cordey, F. Laubscher, M. Docquier, G. Vieille, C. Van Delden, V. Braunersreuther, Mc Kee TA, J. A. Lobrinus, S. Masouridi-Levrat, Y. Chalandon, L. Kaiser, D. L. Vu

**Affiliations:** 1 Division of Infectious Diseases, Geneva University Hospitals, 4 Rue Gabrielle-Perret-Gentil, 1211, 14 Geneva, Switzerland; 2 Laboratory of Virology, Division of Laboratory Medicine, Geneva University Hospitals, 4 Rue Gabrielle-Perret-Gentil, 1211, 14 Geneva, Switzerland; 3 University of Geneva Medical School, Geneva, Switzerland; 4 iGE3 Genomics Platform, University of Geneva, Geneva, Switzerland; 5 Department of Genetics and Evolution, University of Geneva, Geneva, Switzerland; 6 Clinical Pathology Service, Diagnostic Department, Geneva University Hospitals, Geneva, Switzerland; 7 Division of Hematology, Department of Oncology, Geneva University Hospitals, Geneva, Switzerland; 8 Geneva Centre for Emerging Viral Diseases, Geneva, Switzerland

**Keywords:** Viral infection, Metagenomics, Transplantation, Graft-versus-host disease, Rubella, Anellovirus, Human pegivirus, Astrovirus, Polyomavirus, Protoparvovirus

## Abstract

**Background:**

Viral infections are common complications following allogeneic hematopoietic stem cell transplantation (allo-HSCT). Allo-HSCT recipients with steroid-refractory/dependent graft-versus-host disease (GvHD) are highly immunosuppressed and are more vulnerable to infections with weakly pathogenic or commensal viruses. Here, twenty-five adult allo-HSCT recipients from 2016 to 2019 with acute or chronic steroid-refractory/dependent GvHD were enrolled in a prospective cohort at Geneva University Hospitals. We performed metagenomics next-generation sequencing (mNGS) analysis using a validated pipeline and de novo analysis on pooled routine plasma samples collected throughout the period of intensive steroid treatment or second-line GvHD therapy to identify weakly pathogenic, commensal, and unexpected viruses.

**Results:**

Median duration of intensive immunosuppression was 5.1 months (IQR 5.5). GvHD-related mortality rate was 36%. mNGS analysis detected viral nucleotide sequences in 24/25 patients. Sequences of ≥ 3 distinct viruses were detected in 16/25 patients; *Anelloviridae* (24/25) and human pegivirus-1 (9/25) were the most prevalent. In 7 patients with fatal outcomes, viral sequences not assessed by routine investigations were identified with mNGS and confirmed by RT-PCR. These cases included Usutu virus (1), rubella virus (1 vaccine strain and 1 wild-type), novel human astrovirus (HAstV) MLB2 (1), classic HAstV (1), human polyomavirus 6 and 7 (2), cutavirus (1), and bufavirus (1).

**Conclusions:**

Clinically unrecognized viral infections were identified in 28% of highly immunocompromised allo-HSCT recipients with steroid-refractory/dependent GvHD in consecutive samples. These identified viruses have all been previously described in humans, but have poorly understood clinical significance. Rubella virus identification raises the possibility of re-emergence from past infections or vaccinations, or re-infection.

Video abstract

**Supplementary Information:**

The online version contains supplementary material available at 10.1186/s40168-020-00953-3.

## Background

Viral primary infections and reactivations are common complications after allogeneic hematopoietic stem cell transplantation (allo-HSCT) and are associated with significant morbidity and mortality [1, 2]. Current routine clinical surveillance molecular assays detect specific nucleotide sequences targeting usual culprits including Epstein-Barr virus (EBV), cytomegalovirus (CMV), BK polyomavirus (BKPyV), and adenovirus [3]. Metagenomic next-generation sequencing’s (mNGS) unbiased approach broadens viral infection diagnosis, theoretically detecting “all” viral nucleotide sequences or viral infections present [4–7], and is increasingly used in clinical investigations [8]. Allo-HSCT recipients suffering from steroid-refractory/dependent acute or chronic graft-versus-host disease (GvHD) are highly immunosuppressed patients; GvHD immune dysregulation, mucosal barrier alteration [9–13], and multiple prolonged immunosuppressive treatments create a permissive environment for opportunistic viral infections [10, 11, 14]. These clinically unrecognized viral infections can present with limited symptoms/atypical manifestations and lead to intermittent or prolonged viremia [3]. Given the nonspecific clinical features of GvHD and some viral infections, viral infections may remain clinically unrecognized due to limitations of clinical molecular assays.

We hypothesized that some viral infections, which would normally remain undiagnosed with common clinical assays, occur during intense immunosuppressive therapy in steroid-refractory/dependent GvHD. This study aimed to identify viruses that are not routinely searched by RT-PCR routine assays in clinical practice, due to the lack of knowledge. Therefore, we used mNGS on pooled plasma samples of adult allo-HSCT patients with steroid-refractory/dependent acute or chronic GvHD to look for viruses that could be missed by biased technology.

## Methods

### Setting, study population, and design

This observational study was conducted at the Geneva University Hospitals (HUG), Switzerland. The study protocol was approved by the Geneva Cantonal Ethics Commission (project #2019-00511). Inclusion criteria were adult patients who received an allo-HSCT from 1 January 2016 to 31 December 2018 at the HUG, who were enrolled in the local monocentric infectious disease cohort of allo-HSCT patients, and who developed steroid-refractory/dependent acute or chronic GvHD. All included patients provided written consent before enrollment. The only exclusion criteria was the lack of informed written consent.

Plasma samples were prospectively collected during clinical management after allo-HSCT and stored in the Laboratory of Virology, HUG. We performed mNGS analysis on pooled plasma samples of each patient, collected throughout the period of intensive steroid treatment or second-line GvHD therapy.

### Definitions

Steroid-refractory/dependent acute and chronic GvHD were defined according to the position statement of the GvHD experts in Schoemans et al. [15].

### Microbiological methods

#### mNGS and sequence analysis

Each pool (corresponding to 4 to 10 plasma samples from each patient) was prepared to obtain a final volume of 220 μl. Pools were then centrifuged at 10,000×*g* for 10 min to remove cells. Two-hundred microliters of cell-free supernatant were treated with 20 μl of Turbo DNAse (2 U/μl) + 24 μl of 10x TURBO DNase Buffer (Ambion, Rotkreuz, Switzerland), according to the manufacturer’s instructions. Then, the whole volume was divided into two tubes of 120 μl each. One tube was then used for each of the two nucleic acid extraction procedures. Indeed, virus genome extractions were done using two previously published protocols in parallel [16], namely the RNA and DNA protocols optimized for the detection of RNA and DNA viral sequences, respectively.

For the RNA protocol, nucleic acids were extracted with TRIzol (Invitrogen, Carlsbad, CA, USA). Ribosomal RNA was removed (Ribo-Zero Gold depletion kit (Illumina, San Diego, USA) before libraries’ preparation (TruSeq total RNA preparation protocol (Illumina)). Libraries’ concentrations and sizes were analyzed using the Qubit (Life Technologies, Carlsbad, CA, USA) and the 2200 TapeStation instruments (Agilent, SantaClara, CA, USA), respectively. Each library was loaded individually in a single lane on the HiSeq 4000 platform (Illumina) using the 2 × 100-bp protocol with dual-indexing. The mean total number of read pairs obtained per pool was 328,936,594.84 (range 252,670,103 to 385,257,539).

For the DNA protocol, nucleic acids were extracted with the NucliSens easyMAG magnetic bead system (bioMérieux, Geneva, Switzerland). As previously published [16], double-stranded DNA synthesis was done with the DNA polymerase I, Large Fragment (Klenow) (New England BioLabs, Ipswich, MA, USA)). Libraries were prepared using the Nextera XT (Illumina) protocol. Libraries’ concentrations and sizes were analyzed using the Qubit (Life Technologies) and the 2200 TapeStation instruments (Agilent), respectively. Each library was loaded individually in a single lane on the HiSeq 4000 platform (Illumina) using the 2 × 100-bp protocol with dual-indexing. The mean total number of read pairs obtained per pool was 301,393,033.48 (range 122,939,325 to 377,758,795).

To check for potential contaminating viral sequences from environment or experimental reagents [17], four negative controls (i.e., Neg1-4) were submitted to the whole mNGS procedure. To assess the mNGS process efficiency, positive controls underwent the whole mNGS procedure (canine distemper virus (CDV)-spiked samples and a baculovirus (GenScript, Piscataway, NJ, USA) harboring 793 nucleotides of the CDV fusion gene were used as positive controls for the RNA and DNA protocols, respectively).

Paired reads were quality filtered using Trimmomatic [18]. Reads mapped against the human genome and transcriptome (hg38, gencode.V23) were removed using SNAP [19]. Remaining reads were analyzed using two methods in parallel as previously described [20]: (1) by a pipeline that used virusscan 1.0 (https://github.com/sib-swiss/virusscan) to map reads against the Virosaurus database (version V90v_2018_11) (https://viralzone.expasy.org/8676), which is designed to report vertebrate viruses, and (2) by de novo assembly. Only viruses with ≥ 300 nucleotides of coverage were reported. The raw sequence data were deposited in the NCBI Sequence Read Archive under BioProject accession number PRJNA641787.

Sequences were considered *clinically recognized viruses* if they corresponded to a virus known to reactivate in/be frequently found among allo-HSCT recipients [2, 4] or if the patient had a known chronic viral infection and *clinically unrecognized viruses* if not.

#### Confirmatory real-time (reverse transcription-)polymerase chain reaction (r(RT-)PCR) assays

Clinically unrecognized mNGS findings were confirmed on unpooled plasma by specific semi-quantitative or quantitative r(RT-)PCR assays as previously published; additional specimens (including plasma, cerebrospinal fluid, bronchoalveolar lavage (BAL) fluids, nasopharyngeal swabs, native urines, stools suspension, tissue biopsies or bone marrow) were tested when available and pertinent. Quantitative r(RT-)PCR assays were done for Mamastrovirus 1 (classical) using the updated human astrovirus (HAstV) combination [21], Mamastrovirus 6 (MLB2) using the MLB2 assay [22], Usutu virus [23], and bufavirus using the BuV (NS1) assay [24]. Semi-quantitative r(RT-)PCR assays were done for cutavirus using the CuV (VP2) assay [24], human polyomavirus (HPyV) 6 using the VP2 assay [25], HPyV-7 using the VP2 assay [25], and rubella virus [26].

Nucleic acids from plasma, cerebrospinal fluid, BAL fluids, nasopharyngeal swabs, urine, stools resuspended in PBS, and bone marrow were extracted individually from 190 μL of each specimen, spiked with 10 μL of standardized CDV as internal control [27], using the NucliSENS easyMAG (bioMérieux, Geneva, Switzerland) nucleic acid kit, according to the manufacturer’s instructions, and eluted in 25 μL. DNA and RNA were extracted from tissue biopsies using the QIAamp DNA FFPE Tissue Kit (Qiagen, Hombrechtikon, Switzerland) and High Pure FFPET RNA isolation kit (Roche Applied Sciences, Indianapolis, IN, USA), respectively, following the manufacturer’s instructions. For RNA viruses, the rRT-PCR assays were performed using the one-step QuantiTect Probe RT-PCR Kit (Qiagen, Hombrechtikon, Switzerland) in a StepOne Plus instrument (Applied Biosystems, Rotkreuz, Switzerland). For DNA viruses, the rPCR assays were performed using the TaqMan Universal PCR Master Mix (Applied Biosystems) in a StepOne Plus instrument (Applied Biosystems) for cutavirus and bufavirus or in a QuantStudio 5 instrument (Applied Biosystems) for HPyV6 and 7.

For quantitative r(RT)-PCR assays, standard curves and lower limit of quantifications (LOQ) were assessed using 10-fold serial dilutions of specific RNA oligonucleotides (Mamastrovirus 1 (classical) and 6 (MLB2): LOQ = 1.25E4 and 1.25E3 RNA copies/ml of plasma, respectively), RNA transcript (Usutu virus: LOQ = 1.32E2 RNA copies/ml of plasma), DNA oligonucleotides (bufavirus: LOQ = 1.32E3 DNA copies/ml of plasma), or plasmids (HPyV6 and 7: LOQ = 2.63E2 DNA copies/ml of plasma each) containing the target sequences.

#### Statistical analysis

Categorical variables were described by counts and percentages. Continuous variables were expressed as mean and standard deviation or median and interquartile range.

## Results

### Patient characteristics

We identified 25 adult allo-HSCT recipients with acute or chronic steroid-refractory/dependent GvHD. Table 1 shows the patient’s characteristics. The median duration of intensive immunosuppression was 5.1 months (IQR 5.5), and 22/25 patients received ruxolitinib. At the time of writing, fifteen patients have died, with 9 considered as GvHD-related.

**Table 1:** Patients’ characteristics (25 allo-HSCT patients)

	Total
	*n* = 25
**Demographics**	
Sex (male), *n* (%)	16 (64.0)
Age, median (IQR)	58.0 (25.0)
**Allo-HSCT considered in the analysis,** ***n*** **(%)**	
First	23 (92.0)
Second	2 (8.0)
**Transplant source,** ***n*** **(%)**	
Bone marrow	5 (20)
Peripheral blood cells	20 (80)
**Underlying disease,** ***n*** **(%)**	
Acute myeloid leukemia	10 (40.0)
Lymphoid malignancy	6 (24.0)
MDS/MDPS	3 (12.0)
Acute lymphoid leukemia	2 (8.0)
Other^a^	4 (16.0)
**Risk score,** ***n*** **(%)**	
Low	0 (0)
Intermediate	18 (72.0)
High	7 (28.0)
**Donor sex, M,** ***n*** **(%)**	7 (28.0)
**Donor age, median (IQR)**	36 (16.5)
**Donor match,** ***n*** **(%)**	
Donor-related	10 (40.0)
**CMV donor/recipient constellation,** ***n*** **(%)**	
+/+	12 (48.0)
−/+	1 (4.0)
+/−	7 (28.0)
−/−	5 (20.0)
CMV prophylaxis, *n* (%)	1 (4.0)
**Conditioning,** ***n*** **(%)**	
Myeloablative conditioning	5 (20.0)
**GvHD prophylaxis,** ***n*** **(%)**	
Calcineurin inhibitor	24 (96.0)
Mycophenolate mofetil	17 (68.0)
Methotrexate	7 (28.0)
**GvHD organ,** ***n*** **(%)**	
Digestive tract	17 (68.0)
Skin	15 (60.0)
Mouth	4 (16.0)
Liver	7 (28.0)
Lung	5 (20.0)
Eyes	2 (8.0)
Musculoskeletal	1 (4.0)
**GvHD grade or severity,** ***n*** **(%)**	
Grade	
2	9 (36.0)
3	3 (12.0)
4	6 (24.0)
Moderate	6 (24.0)
Severe	7 (28.0)
**GvHD treatment,** ***n*** **(%)**	
Corticosteroids	24 (96.0)
Ruxolitinib	22 (88.0)
Calcineurin inhibitor	21 (84.0)
Mycophenolate mofetil	12 (48.0)
Photopheresis	12 (48.0)
Budenoside	5 (20.0)
Sirolimus	3 (12.0)
Tocilizumab	2 (8.0)
Other^b^	6 (24.0)
**Death,** ***n*** **(%)**	15 (60.0)
Median delay from allo-HSCT, months (IQR)	11.2 (15.8)

One patient could have multiple GvHD prophylaxis and treatment, multiple organs with GvHD and multiple grades of severity. CMV prophylaxis: one patient (patient Ge24) received letermovir during the period of intensive steroid treatment or second-line GvHD therapy. GvHD grade refers to acute GvHD, GvHD severity refers to chronic GvHD. In two patients, there was no information on grade/severity. Only organs with grade GvHD ≥ 2 or severity ≥ moderate are reported

*Abbreviations*: *IQR* interquartile range, *allo-HSCT* allogeneic hematopoietic stem cell transplantation, *MDS/MDPS* myelodysplasic syndrome/myelodysplasic proliferative syndrome, *ATG* anti-thymocyte globulin

^a^Other includes: multiple myeloma (*n* = 2), chronic myeloid leukemia (*n* = 1) and mixed acute leukemia (*n* = 1)

^b^Other includes: azithromycine, montelukast, prolastin, vedolisumab, nilotinib, basilixumab, ibrutinib, and/or methotrexate

### Viral sequences identified with mNGS and confirmatory analyses

**Fig. 1:**
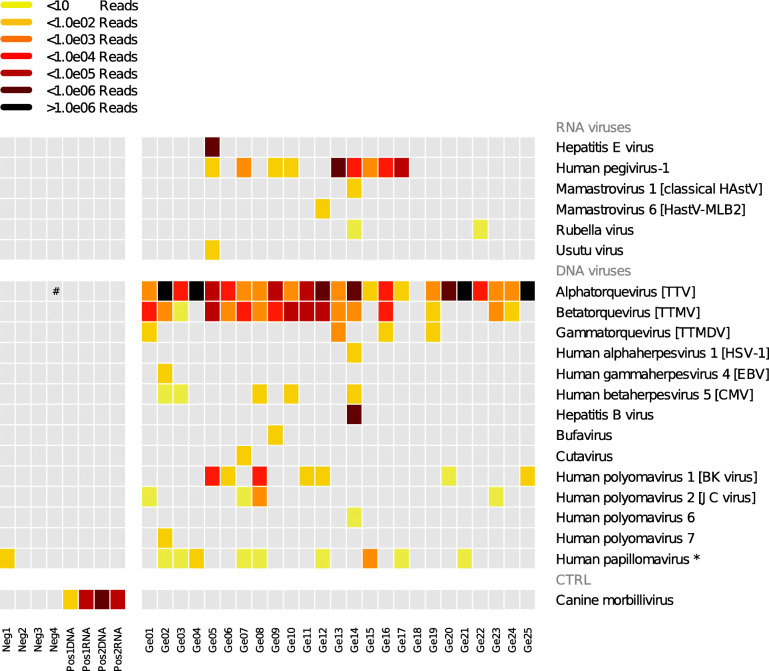
Grid plot of viral sequences identified with mNGS in pooled plasma samples (25 allo-HSCT patients). Each line corresponds to viral sequences assigned to one virus; the bottom line corresponds to mNGS negative (Neg1-4) and positive (Pos1/2 DNA/RNA) control samples. Each column corresponds to one pool of plasma sample (one patient). Colors represent the approximate number of reads matching virus genome detected in each pool of plasma samples. *Since they were detected in one negative control, human papillomavirus sequences were considered as likely contaminant. # cross-contamination. *Abbreviations*: *HAstV* human astrovirus, *TTV* torque teno virus, *TTMV* torque teno minivirus, *TTMDV* torque teno midivirus, *HSV-1* herpes simplex 1 virus, *EBV* Epstein-Barr virus, *CMV* cytomegalovirus, *CTRL* control

**Fig. 2:**
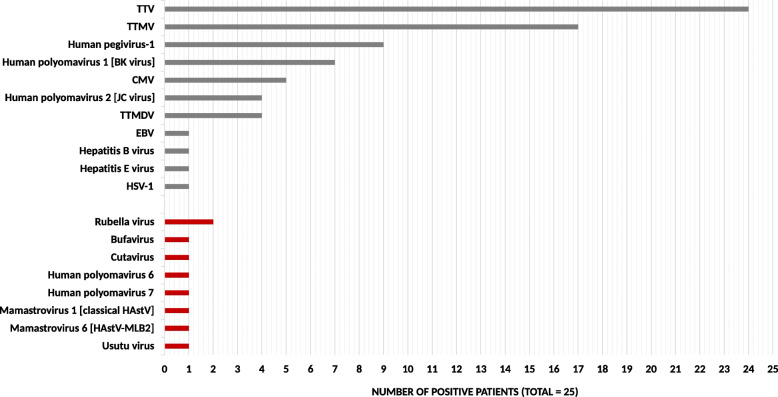
Prevalence of viruses identified with mNGS in pooled plasma samples (25 allo-HSCT recipients). The vertical axis represents all identified viral sequences. The horizontal axis represents the number of patients in which sequences of each virus were identified. *Abbreviations*: *TTV* torque teno virus, *TTMV* torque teno minivirus, *CMV* cytomegalovirus, *TTMDV* torque teno midivirus, *EBV* Epstein-Barr virus, *HSV-1* herpes simplex 1 virus, *HAstV* human astrovirus

The mNGS analysis revealed viral nucleotide sequences in all patients except Ge18 (24/25). In 16/25 patients, ≥ 3 distinct viral species were detected (Fig. 1). Figure 2 depicts the prevalence of each identified virus, and Table S1 provides detailed mNGS characteristics.

*Anelloviridae* (torque teno virus (TTV), torque teno minivirus (TTMV) and torque teno midivirus (TTMDV)) and human pegivirus-1 (HPgV-1) were the most prevalent with sequences detected in 24/25 and 9/25 patients, respectively.

Other detected DNA viruses included BK polyomavirus (BKPyV) (7/25), CMV (5/25), and JC polyomavirus (JCPyV) (4/25), and also herpes simplex virus 1 (HSV-1), EBV, hepatitis B virus (HBV), bufavirus, cutavirus, and HPyV-6/7, each detected once (1/25). Except HPgV-1, the detected RNA viruses were less prevalent than DNA viruses and were HAstV (Mamastrovirus 1 and 6) and rubella virus, both detected twice (2/25), and also hepatitis E (HEV) and Usutu viruses, both detected once (1/25). The de novo analysis did not reveal other relevant sequences. Interestingly, sequences for TTV (11/24), CMV (3/5), EBV (1/1), and HBV (1/1) were detected in both DNA and RNA libraries, suggesting active viral replication (Figure S1).

### Clinically recognized viral sequences

Although not routinely searched in clinical practice, *Anelloviridae* and HPgV-1 sequences were identified in 96% and 36% of patients and were classified among clinically recognized viral sequences as they are known to be highly prevalent among immunocompromised patients. In 14/25 patients, mNGS analysis identified sequences of latent DNA viruses known to reactivate in transplant recipients (EBV, CMV, HSV-1, BKPyV, JCPyV, HBV). At the RNA level, HEV was identified once.

The r(RT-)PCR assays performed during routine investigations confirmed the mNGS analysis (Table S2): patient Ge05 had a chronic HEV infection, and patient Ge14 had chronic HBV and cutaneous HSV-1 infections, and HSV-1 viremia. BKPyV and JCPyV were not systematically screened and were only revealed by mNGS analysis. However, BKPyV was screened in patient Ge06 and detected at low viral loads (VL) (1.41E2 and 1.44E3 copies/ml) in two plasma samples collected a few days apart from those included for mNGS analysis. In 12/25 patients, CMV and EBV were detected only by rPCR at low VL; no other expected virus was detected by routine molecular assays.

### Clinically unrecognized viral sequences

We found clinically unrecognized sequences belonging to either rare and/or recently identified viruses (HAstV MLB2, Usutu virus, bufavirus, cutavirus, HPyV-6, and HPyV-7) or those not routinely assessed alongside GvHD (classic HAstV and rubella virus) in 7/25 patients, whose characteristics are detailed in Table 2.

**Table 2:** Clinical characteristics of 7 patients with clinically unrecognized viral sequences

Patient’s code	Age, gender	Underlying disease	GvHD organ	GvHD treatments^a^	Outcome
**Ge02**	61, M	Lymphoma	Digestive, skin	CSA, tacrolimus, corticosteroids, sirolimus, budenoside, photopheresis	Death (GvHD-related)
**Ge05**	23, M	Lymphoma	Digestive, skin, lung	CSA, MMF, tacrolimus, corticosteroids	Death
**Ge07**	60, M	ALL	Digestive	Tacrolimus, corticosteroids, photopheresis	Death
**Ge09**	65, M	AML	Liver, digestive^b^	CSA, corticosteroids, tocilizumab	Death
**Ge12**	44, M	MM	Digestive, liver	CSA, corticosteroids, basiliximab, MMF	Death
**Ge14**	31, M	AML	Digestive, liver	Corticosteroids	Death
**Ge22**	68, M	MDPS	Digestive, skin	CSA, tacrolimus, corticosteroids, vedolisumab, prolastin	Death

Age at the time of transplantation

*Abbreviations*: *GvHD* graft-versus-host disease, *M* male, *ALL* acute lymphoid leukemia, *AML* acute myeloid leukemia, *MM* multiple myeloma, *MDPS* myelodysplasic proliferative syndrome, *CSA* cyclosporine A, *MMF* mycophenolate mofetil

^a^All patients had ruxolitinib as part of the GvHD treatment

^b^Digestive GvHD was not confirmed by biopsies

mNGS identifications of these clinically unrecognized viral sequences were confirmed by r(RT-)PCR in unpooled plasma samples. Whenever available during/after the period of sample selection, additional specimens and/or tissue biopsies were screened over a median period of 7.1 weeks (IQR 25.3). These findings are shown in Table 3.

**Table 3:** mNGS and r(RT-)PCR results of 7 patients with clinically unrecognized viral sequences

	Viral species detected with mNGS	Viral species confirmed with r(RT)-PCR assays			
Patient’s code	Virus	Plasma, ***n*** (pos/total)	Time period (days)	Estimated viral load (mean log10 copies/ml plasma or ***CT values***)	Other positive biological specimen
**Ge02**	HPyV7	14/14	279	4.75	BM, BAL
**Ge05**	Usutu virus	2/5	7	4.66	–
**Ge07**	Cutavirus	6/10	98	*37.9*	Skin
**Ge09**	Bufavirus	6/12	81	3.73	Stools, duodenum
**Ge12**	Novel human astrovirus MLB2	2/6	14	3.24	Intestine, colon, BM
**Ge14**	Rubella virus	3/5	39	*37.9*	–
	Classic human astrovirus	5/11	25	5.56	–
	HPyV6	11/11	74	3.76	–
**Ge22**	Rubella virus	2/8	3	*36.7*	–

Pos/total corresponds to the number of positive samples on the total of screened samples. Time period corresponds to the time period during which samplings were found positive. CT values above 40 were considered negative. CT values are indicated in *italics*

*Abbreviations*: *BM* bone marrow, *BAL* bronchoalveolar lavage, *CT* cycle threshold

### Brief clinical description of patients harboring clinically unrecognized viral sequences

#### Ge02: HPyV-7

A 61-year-old male, transplanted for lymphoma, developed digestive and skin GvHD; intensive immunosuppression for GvHD lasted from 12 to 16 months after transplantation (4-month sample period), when the patient died from a post-transplant EBV-related lymphoma disease. Beginning 9 months before death, we found HPyV-7 in all screened plasma samples. In addition, HPyV-7 was also found in a BAL and a bone marrow sample collected 8 months after transplantation (2.8 log10 DNA copies/ml and CT value 28, respectively).

#### Ge05: Usutu virus

A 23-year-old male, transplanted for lymphoma, developed digestive skin and lung GvHD; intensive immunosuppression for GvHD lasted from 1 to 24 months after transplantation (22-month sample period), when the patient died from a disseminated fungal infection and GvHD. We found Usutu virus in 2 plasma samples collected 7 days apart, a few days after a blood transfusion, and 1.5 months before death.

#### Ge07: cutavirus

A 60-year-old male, transplanted for acute lymphoblastic leukemia, developed digestive GvHD; intensive immunosuppression for GvHD lasted from 6 to 8 months after transplantation (2-month sample period), when the patient died from a disseminated fungal infection and GvHD. We found cutavirus in a skin biopsy performed 3 weeks before transplantation (CT of 35) and at low plasma VL for 3 months before death. Autopsy results confirmed digestive GvHD and a disseminated mold infection.

#### Ge09: bufavirus

A 65-year-old male, transplanted for acute myeloid leukemia (AML), developed liver GvHD; intensive immunosuppression for GvHD lasted from 5 to 7 months after transplantation (2 month sample period), when the patient died from GvHD. We detected bufavirus RNA in several plasma samples taken over 2 months before death. In addition, bufavirus was also found in stool samples collected the day of and 1.5 months after transplantation, at CT values of 38.2 and 34.7, respectively, and in a duodenal biopsy performed 5 months after transplantation (CT value 27.3), which revealed chronic duodenitis.

#### Ge12: HAstV MLB2

A 44-year-old male, transplanted for multiple myeloma, developed a digestive and liver GvHD; intensive immunosuppression for GvHD lasted from 15 days to 2 months after transplantation (2-month sample period), when the patient died from the GvHD. We detected HAstV MLB2 RNA in two plasma samples collected 14 days apart and shortly before death, in a colonic biopsy performed few days before death (CT value 27.9), and in several intestine, colonic, and bone marrow autopsy samples (mean CT values 25.8, 27.6, 28, respectively). Autopsy results confirmed liver and digestive GvHD and revealed chronic pulmonary GvHD.

#### Ge14: rubella virus, classic HAstV, HPyV-6

A 31-year-old male of Chinese origin, transplanted for AML, developed digestive and liver GvHD; intensive immunosuppression for GvHD lasted 9–11 months after transplantation (3-month sample period), when the patient died from an acute intestinal perforation in the context of recurrent digestive GvHD. In several plasma samples starting 1 month before death, we found classic HAstV RNA and low VLs of a wild-type Chinese rubella strain. HPyV-6 DNA was also persistently found in plasma samples starting 2 months before death. The patient was seropositive for rubella before transplantation.

#### Ge22: rubella virus

A 68-year-old Portuguese male, transplanted for myelodysplastic syndrome, developed digestive and skin GvHD; intensive immunosuppression for GvHD lasted from 4 to 6 months after transplantation (6-week sample period), when the patient died from GvHD. Two plasma samples were positive for rubella-vaccine RNA at low VLs. The patient was seropositive before transplantation.

## Discussion

We analyzed viral sequences by mNGS in pooled plasma samples of 25 adult allo-HSCT patients with severe steroid-refractory/dependent GvHD. Viral nucleotide sequences were found in 96% of patients, and 64% of patients had ≥ 3 distinct viral species. Besides commensal (*Anelloviridae* and HPgV-1) and latent (EBV, CMV, HSV-1, BKPyV, JCPyV) viruses known to be highly prevalent/reactivate in allo-HSCT recipients, 28% of patients had clinically unrecognized viral sequences that are rarely/never reported in allo-HSCT patients, with unknown pathogenicity (bufavirus, cutavirus, HPyV-6/7, novel HAstV-MLB2, classic HAstV, rubella virus, and Usutu virus). Chronic HBV and HEV infections were also confirmed by mNGS in two patients.

*Anelloviridae* (TTV, TTMV, and TTMDV) were the most prevalent in this study, concordant with the high TTV viremia rate identified among allo-HSCT recipients [28–31]. The high TTV, TTMV, and TTMDV co-detection rate has been previously described [30]. Chronic anelloviruses infection/re-infection is common, but disease associations remain undetermined [32]. Among allo-HSCT recipients, two studies failed to demonstrate any association between TTV viremia and immune-related complication or other viral reactivations [28, 33], while a third reported higher TTV viremia in patients receiving corticosteroids for GvHD [29]. A recent study found higher TTV VL at 100 days post-transplantation predicts worse overall survival, and a higher risk of acute GvHD and infections [34]. Finally, a mNGS study revealed increased detection rates and number of *Anelloviridae* sequences in stool samples of allo-HSCT recipients several weeks after developing digestive GvHD [5], suggesting a consequence of GvHD-associated inflammation and/or immunosuppressive therapy. Altogether, these data suggest that TTV viremia could be a potential immunosuppression-marker, requiring further investigation.

HPgV-1 (a *Flaviviridae* [35]) viremia occurs in 1–4% of blood donors in developed countries [36] and up to 30% of allo-HSCT recipients but has not been associated with clinical consequences [3, 4]. Given the interaction of HPgV-1 with the immune system [37], the effect of persistent HPgV-1 viremia requires deeper investigation.

We found four species of HPyV (JCPyV, BKPyV, HPyV-6/7), with JCPyV viremia occurring in 16% of allo-HSCT recipients, agreeing with studies where concomitant use of multiple immunosuppressive treatments was associated with increased persistent viremia risk—although progressive multifocal leucoencephalopathy was rare [38]. In another study, JCPyV DNA detection rate in plasma decreased from 4/22 to 1/22 patients at 3 and 12–18 months after transplantation, respectively, while viremia was not linked to any clinical manifestation [39]. Our study’s BKPyV prevalence (28%) was lower than the 54% of another study [40]. Notably, our patients did not develop hemorrhagic cystitis.

Contamination or other bioinformatics errors were excluded for each of the clinically unrecognized viral sequences, by confirming the mNGS-identified viral sequences using r(RT-)PCR on blood and non-blood samples at different time-points. These assays found cutavirus (60%) and bufavirus (50%), two *Protoparvoviruses*, in plasma samples at low VL. Interestingly, available skin and digestive biopsy tissues, previously described as putative primary infection sites [24, 41, 42], were positive by PCR at or shortly before transplantation, indicating viral latency and reactivation under immunosuppressive conditions. Bufavirus was first discovered in stool samples of children in 2012, and the stool prevalence is about 0.3–4.1%, although high seroprevalence was identified in some countries [41, 42]. Bufavirus was recovered in stools of subjects with digestive symptoms, but not in asymptomatic controls [41]. Whether bufavirus influences syndromes attributed to digestive GvHD remains unknown. Cutavirus was discovered in 2016 in stools of diarrheic children in Brazil with a prevalence of about 1–1.6%, and curiously, some associations were made with cutaneous T cell lymphoma [24, 41]. To our knowledge, cutavirus and bufavirus viremia have not been described before.

Usutu virus (a *Flaviviridae*) is an arbovirus, endemic in Africa and several European countries, that frequently co-circulates with West Nile virus [43–45]. The virus enzootic cycle involves birds (main reservoir) and ornithophilic mosquitos (vectors); humans are incidental and dead-end hosts [43]. Less than 50 documented cases of acute Usutu virus infections have been reported in humans, most of them corresponding to the identification of Usutu virus genome in donated blood samples [45]. Human Usutu virus infection can be asymptomatic or associated with various clinical manifestations, including fever, rash, and neurological manifestations; the virus genome was detected in some cases in CSF or blood samples [43, 45, 46]. The virus can infect neurons, astrocytes, microglia cells, and induced pluripotent stem cell (IPSc)-derived human neuronal stem cells, with a reduction in cell proliferation, induction of antiviral response, and apoptosis [47]. In our study, Usutu virus was transiently identified shortly after a blood transfusion, but retrospective blood bag testing was impossible. Although blood transmission has not been described, screening over ≥ 130,000 blood donations revealed 38 positive donors [48, 49]. Although overt clinical consequences are absent in our patient, this flavivirus is known to cause occasional complications [45] and cannot be disregarded. If transmission occurred by transfusion, it is possible that only remnant RNA was transmitted, not infectious virus.

Astroviruses are well-recognized enteric viruses infecting mainly children, elderly, and immunocompromised patients [50]. HAstV MLB2 was identified a decade ago [51], and since then, it has been demonstrated that it is circulating in every continent [50]. In addition, it is associated with systemic and central nervous system infections [7, 52] and has been identified in stool samples of asymptomatic children [53]. In our mNGS study, classic HAstV was found in plasma samples of a patient with digestive GvHD shortly before death, and HAstV MLB2 was found at low VL in plasma samples of a patient with diarrhea due to digestive GvHD. In the latter, autopsy confirmed the presence of HAstV MLB2 RNA in several intestinal and colonic samples at significant VLs. The presence of astrovirus MLB2 in the digestive tract of the patient is thus evident, and HAstV viremia plausibly came from an intestinal spillover in the context of the GvHD and intestinal perforation.

HPyV-6 and 7 were discovered in 2010 [54] and have been identified in a wide range of clinical samples of healthy subjects, transplant recipients, and symptomatic immunocompetent patients [55–59]. They have been detected mostly in skin specimens of non-transplanted individuals and transplanted recipients with or without dermatological diseases, but the association with clinical manifestation is not established [55, 57, 59–61]. The reported seroprevalence rates of HPyV6 and HPyV7 in immunocompetent and immunocompromised adults varies from 69 to 84% and 35 to 72%, respectively [3]. The transmission route, tropism, pathogenic mechanisms, and potential association with human diseases are still not established. HPyV-6 DNA prevalence in healthy blood donors is 0.1% and 0.6% in kidney transplant recipients [3], while HPyV-7 has been detected in lung transplant recipients [3], yet no obvious clinical manifestations are associated with them. We report persistent HPyV-6/7 viremia in plasma samples of 2/25 allo-HSCT recipients over several months. Further investigations are needed to determine their pathogenicity.

Rubella virus was our most unexpected finding, yet the rubella reads for both patients mapped to different regions along the genome (rubella virus genome coverage of 3.69% and 5.47% for patients Ge14 and Ge22, respectively) and mNGS results were confirmed by specific rRT-PCR in several samples over a period of 39 and 3 days for patients Ge14 and Ge22, respectively.

Although rubella can persist in in vitro and animal models [62–64], it is not known to persist after vaccination or natural infection in humans, except in vaccinated immuno-deficient children [65–68]. Chronic rubella infection has also been hypothesized as causing Fuch’s heterochromic iridocyclitis, although the pathophysiology remains unknown [69, 70]. We found rubella sequences with low VLs in two patients, each with a distinct strain: a vaccine strain and a Chinese strain that was found in a patient who previously lived in China. Macrophages and keratinocytes are potential sites for rubella persistence [66], but retrospectively screened skin samples from one patient gave negative results. Both patients were seropositive before transplantation. Patients with GvHD frequently become seronegative for measles and rubella within 2 years after allo-HSCT [71]. Identification of the usual vaccine strain and a Chinese strain in a native Chinese, strongly argues for reactivation, in line with antibody loss after transplantation. Yet, we noted a decreased rubella IgG titer in one of both patients at the time of viremia, compared to the pre-transplant titers. Gonzalez et al. reported the case of a child who developed fulminant hepatitis after stem cell transplantation despite prior vaccination [72]. The hypothesis raised by the authors of a primary infection from a recently vaccinated contact implies that circulating vaccine strains in the population could be an issue for immunocompromised patients with waning antibodies. Whether rubella persistence could trigger GvHD after transplantation and where the viral reservoir would be are open questions.

A recent trial identified ruxolitinib as a second-line treatment for steroid-refractory GvHD, which has a poor prognosis and no approved clearly beneficial treatment [14, 73]: in the study, about 1/3 patients experienced a grade 3 infection, highlighting the importance of monitoring patients for infections. Our study reveals that some viral infections were overlooked by standard procedures, which may indicate that the 30% of infection risk associated with ruxolitinib could be underestimated, and raises the question of including mNGS analysis in the management of high-risk patients.

Among the 7 patients with clinically unrecognized viral sequences, most sequences were identified a few weeks before and, persisted until, patients’ deaths. Although neither the pathogenic nature of the viruses nor the clear associations with patient outcomes are proven, the identification of these viral sequences in patients’ blood during severe GvHD is relevant and reflects the altered immune response; monitoring these infections could help adjust immunosuppressive therapies. Among these patients, with nearly daily blood sample collection, such strategies could routinely be actionable by pooling plasma samples (overcoming transient viremia problems), with the aim of excluding disseminated infections before increasing immunosuppression, and unmasking a viral infection mimicking the GvHD syndrome.

The identification of HAstV and bufavirus in digestive tracts of patients with digestive GvHD may merely reflect the patient’s gut virome, but identification of enteric viruses in blood samples could indicate a disseminated infection that is triggered by GvHD inflammation or immunosuppressive treatment, which may require treatment adaptation.

These viral infections cannot be considered innocent bystanders. Most of the identified viruses can be shed asymptomatically, but certainly lead to organ disease under conditions where they become opportunistic pathogens, potentially causing unrecognized clinical features; they can also lead to a clinical exacerbation. The particular immunologic state of our population may influence this delicate balance between an indolent virus and its clinical impact.

A major limitation of this study is the small monocentric cohort. Additionally, including only allo-HSCT recipients with steroid-refractory/dependent GvHD precluded generalization of the results to all allo-HSCT recipients. Furthermore, we lacked control patients without GvHD. Despite the numerous viral infections revealed by mNGS, whether these are specific to patients with GvHD or to those treated with ruxolitinib, and if there is an association with clinical manifestations and/or an impact on the immune state of these patients, remains to be determined by appropriate studies. Notably, according to the comparison with routine diagnostic results and confirmatory r(RT-)PCR, our mNGS pipeline is accurate, although with a lower sensitivity compared to specific quantitative real-time PCR assays used in most routine laboratories.

## Conclusions

Blood analysis of patients with steroid-refractory/dependent GvHD revealed clinically unrecognized viral sequences in 28% of patients, including rubella virus, novel protoparvoviruses, HPyV-6/7, Usutu virus, and HAstV-MLB2. These viruses have been described in humans, but rarely reported as causes of disease in allo-HSCT patients, or have unknown pathogenicity. Rubella virus identifications imply possible re-emergence from past infection or vaccination. Further investigations are needed to understand the clinical significance of these infections.

## Supplementary Information

**Additional file 1: Table S1.** Detailed mNGS results per patient for the 25 adult allo-HSCT patients. **Table S2.** Comparison of mNGS results on the pooled plasma samples and of the routine r(RT-)PCR results on the corresponding plasma samples of the 25 patients. **Figure S1.** Boxplot of mapped reads of DNA viruses and corresponding mRNA detection with mNGS. The vertical axis represents the number of mapped reads. The horizontal axis represents all identified viral sequences of DNA viruses (left panel) and corresponding mRNA sequences (right panel). The numbers on the horizontal axis represent the number of patients in which sequences of each virus were identified. Abbreviations: HSV-1: herpes simplex 1 virus; CMV: cytomegalovirus; EBV: Epstein-Barr virus; TTMDV: torque teno midivirus; TTMV: torque teno minivirus; TTV: torque teno virus.

## Data Availability

The datasets generated and/or analyzed during the current study are available in the Dryad repository (10.5061/dryad.0k6djh9xp). The raw sequence data were deposited in the NCBI Sequence Read Archive under BioProject accession number PRJNA641787.
