# Supplementary material for: Unmasking viral sequences by metagenomic next-generation sequencing in adult human blood samples during steroid-refractory/dependent graft-versus-host disease
Source: Microbiome. 2021 Jan 24;9:28. doi: 10.1186/s40168-020-00953-3 (PMC7831233; doi:10.1186/s40168-020-00953-3)

**Additional files**

**Table S1:** Detailed mNGS results per patient for the 25 adult allo-HSCT patients.

| **Patient code** | **Total Paired Reads** | **Virus** | **Mapped reads** | | | **Genome coverage** | **Accession number** |
| --- | --- | --- | --- | --- | --- | --- | --- |
|  |  |  | **Total** | **DNA protocol** | **RNA protocol** |  |  |
| Ge-01 | 678111870 | Human polyomavirus 2 [JC virus] | 4 | 4 | - | 300 | JF424865 |
|  |  | TTMDV | 27 | 27 | - | 754 | AB303566 |
|  |  | TTV | 347 | 347 | - | 3105 | FR751499 |
|  |  | TTMV | 1154 | 1154 | - | 2516 | KP343847 |
| Ge-02 | 741078386 | TTV | 4750432 | 4750297 | 135 | 3286 | KF545578 |
|  |  | TTMV | 418 | 418 | - | 444 | JX134046 |
|  |  | Human betaherpesvirus 5 [CMV] | 12 | 12 | - | 807 | NA |
|  |  | Human gammaherpesvirus 4 [EBV] | 232 | 209 | 23 | 13119 | NA |
|  |  | Human polyomavirus 7 | 68 | 68 | - | 3296 | KJ733012 |
|  |  | Human papillomavirus | 6 | 6 | - | 353 | KC113191 |
| Ge-03 | 705677132 | Human betaherpesvirus 5 [CMV] | 9 | 9 | - | 531 | NA |
|  |  | Human papillomavirus | 8 | 8 | - | 564 | KC113191 |
|  |  | TTV | 1789 | 1600 | 189 | 3452 | KJ194503 |
|  |  | TTMV | 6 | 6 | - | 508 | KP343847 |
| Ge-04 | 677400701 | TTV | 2209978 | 2209947 | 31 | 3401 | FR751490 |
|  |  | Human papillomavirus | 15 | 15 | - | 1046 | U31781 |
| Ge-05 | 659575822 | TTMV | 16133 | 16133 | - | 2699 | KP343847 |
|  |  | TTV | 23282 | 23275 | 7 | 3545 | FR751490 |
|  |  | Human polyomavirus 1 [BK virus] | 9036 | 9036 | - | 5040 | KP984526 |
|  |  | Usutu virus | 26 | - | 26 | 1836 | KY426757 |
|  |  | Hepatitis E virus | 183322 | - | 183322 | 6986 | LC055972 |
|  |  | Human pegivirus-1 | 22 | - | 22 | 1061 | JN127373 |
| Ge-06 | 633025528 | Human polyomavirus 1 [BK virus] | 48 | 48 | - | 2704 | AB485699 |
|  |  | TTMV | 282 | 282 | - | 2420 | KP343847 |
|  |  | TTV | 1597 | 1597 | - | 3248 | KP343839 |
| Ge-07 | 659484293 | Human papillomavirus | 5 | 5 | - | 314 | MF588686 |
|  |  | Human polyomavirus 2 [JC virus] | 7 | 7 | - | 519 | LT615220 |
|  |  | TTV | 368 | 368 | - | 2829 | KT163916 |
|  |  | TTMV | 5223 | 5223 | - | 1210 | JX134046 |
|  |  | Human pegivirus-1 | 661 | - | 661 | 5100 | AF121950 |
|  |  | Cutavirus | 31 | 31 | - | 1733 | KT868811 |
| Ge-08 | 612948233 | TTV | 988 | 988 | - | 3285 | FR751466 |
|  |  | Human polyomavirus 1 [BK virus] | 4793 | 4793 | - | 5212 | AB269824 |
|  |  | Human papillomavirus | 4 | 4 | - | 371 | FJ947080 |
|  |  | Human polyomavirus 2 [JC virus] | 129 | 129 | - | 3952 | LT615220 |
|  |  | TTMV | 730 | 730 | - | 796 | NC_014089 |
|  |  | Human betaherpesvirus 5 [CMV] | 1129 | 1105 | 24 | 55877 | NA |
| Ge-09 | 635701292 | TTV | 77648 | 77569 | 79 | 3544 | FR751499 |
|  |  | TTMV | 5822 | 5822 | - | 2646 | KP343847 |
|  |  | Bufavirus | 16 | 16 | - | 942 | JQ918261 |
|  |  | Human pegivirus-1 | 10 | - | 10 | 581 | KP259281 |
| Ge-10 | 669094598 | TTMV | 13187 | 13187 | - | 2618 | KP343847 |
|  |  | Human betaherpesvirus 5 [CMV] | 97 | 89 | 8 | 4713 | NA |
|  |  | TTV | 640 | 527 | 113 | 3084 | AY449524 |
|  |  | Human pegivirus-1 | 27 | - | 27 | 1366 | AF121950 |
| Ge-11 | 710938991 | Human polyomavirus 1 [BK virus] | 33 | 33 | - | 2056 | AB269824 |
|  |  | TTMV | 19088 | 19088 | - | 2608 | KP343847 |
|  |  | TTV | 37202 | 37202 | - | 3337 | KM593802 |
| Ge-12 | 626989411 | TTMV | 33717 | 33717 | - | 2694 | KP343847 |
|  |  | TTV | 450244 | 450034 | 210 | 3639 | FR848323 |
|  |  | Human polyomavirus 1 [BK virus] | 12 | 12 | - | 1056 | AB485697 |
|  |  | Human papillomavirus | 6 | 6 | - | 341 | X74466 |
|  |  | Mamastrovirus 6 [HAstV-MLB2] | 44 | - | 44 | 1829 | AB829252 |
| Ge-13 | 445133701 | TTV | 116 | 116 | - | 2129 | KP343841 |
|  |  | TTMV | 124 | 124 | - | 1954 | EF538883 |
|  |  | TTMDV | 225 | 225 | - | 401 | AB303565 |
|  |  | Human pegivirus-1 | 357719 | - | 357719 | 8746 | AF121950 |
| Ge-14 | 578285928 | Hepatitis B virus | 136490 | 38 | 136452 | 3951 | AB819611 |
|  |  | Human betaherpesvirus 5 [CMV] | 95 | 69 | 26 | 3457 | NA |
|  |  | TTV | 161532 | 161517 | 15 | 3364 | KJ082064 |
|  |  | TTMV | 972 | 972 | - | 2244 | NC_030297 |
|  |  | Human alphaherpesvirus 1 [HSV-1] | 11 | 11 | - | 737 | NA |
|  |  | Human pegivirus-1 | 1230 | - | 1230 | 7156 | AB008335 |
|  |  | Mamastrovirus 1 [Classical HAstV] | 59 | - | 59 | 2550 | HQ398856 |
|  |  | Rubella virus | 5 | - | 5 | 361 | AY258322 |
| Ge-15 | 609595397 | TTV | 46 | 46 | - | 1768 | AM712003 |
|  |  | Human papillomavirus | 130 | 130 | - | 2079 | NC_001457 |
|  |  | Human pegivirus-1 | 368 | - | 368 | 5424 | AF121950 |
| Ge-16 | 494542789 | TTV | 1053 | 1053 | - | 3511 | AB064596 |
|  |  | TTMDV | 41 | 41 | - | 422 | AB303553 |
|  |  | TTMV | 9945 | 9945 | - | 2632 | KP343847 |
|  |  | Human pegivirus-1 | 1148 | - | 1148 | 6529 | AF121950 |
| Ge-17 | 673096287 | Human papillomavirus | 9 | 9 | - | 300 | KY242583 |
|  |  | Human pegivirus-1 | 19015 | - | 19015 | 8542 | AF104403 |
|  |  | TTV | 63 | 63 | - | 1835 | FR751466 |
| Ge-18 | 503200627 | - | - | - | - | - | - |
| Ge-19 | 508690364 | TTMDV | 18 | 18 | - | 373 | AB303565 |
|  |  | TTV | 664 | 664 | - | 3205 | AF122918 |
|  |  | TTMV | 18 | 18 | - | 753 | KP343847 |
| Ge-20 | 600002119 | TTV | 118540 | 118540 | - | 3162 | KM593802 |
|  |  | Human polyomavirus 1 [BK virus] | 7 | 7 | - | 351 | AB485697 |
| Ge-21 | 753982733 | TTV | 5325950 | 5325803 | 147 | 3319 | NC_014074 |
|  |  | Human papillomavirus | 8 | 8 | - | 330 | KY242583 |
| Ge-22 | 524011379 | TTV | 2579 | 2579 | - | 3526 | FR751497 |
|  |  | Rubella virus | 9 | - | 9 | 535 | AY258322 |
| Ge-23 | 732336028 | TTV | 421 | 414 | 7 | 3508 | FR751497 |
|  |  | Human polyomavirus 2 [JC virus] | 6 | 6 | - | 366 | AB103402 |
|  |  | TTMV | 190 | 190 | - | 2006 | KP343847 |
| Ge-24 | 636885797 | TTMV | 20 | 20 | - | 1202 | KP343847 |
|  |  | TTV | 557 | 557 | - | 3232 | FR751490 |
| Ge-25 | 688451302 | TTV | 1478332 | 1478294 | 38 | 3287 | KF545586 |
|  |  | Human polyomavirus 1 [BK virus] | 22 | 22 | - | 1358 | AB485699 |

Abbreviations: TTV: torque teno virus; TTMV: torque teno minivirus; TTMDV: torque teno midivirus; CMV: cytomegalovirus; EBV: Epstein-Barr virus; HAstV: human astrovirus; HSV-1: herpes simplex 1 virus.

**Table S2 :** Comparison of mNGS results on the pooled plasma samples and of the routine r(RT-)PCR results on the corresponding plasma samples of the 25 patients.

|  | **Viral species detected with mNGS** | | | | **Viral species detected with routine assays only**  **(mean viral load ^†^)** |
| --- | --- | --- | --- | --- | --- |
| **GeBVir Code** | | **Name** |  | **Screening and detection with r(RT)-PCR assays**  **(mean viral load ^†^)** |  |
| **Ge01** | | JCPyV |  | no | CMV (1.92E2 copies/ml) |
| **Ge02** | | EBV |  | yes (1.19E6 copies/ml) | none |
|  | | CMV |  | yes (1.45E2 copies/ml) |  |
| **Ge03** | | CMV |  | yes (4.05E2 copies/ml) | none |
| **Ge04** | | none |  | - | CMV (1.16E2 copies/ml) |
| **Ge05** | | BKPyV |  | no | none |
|  | | HEV |  | yes (CT value 14.3) * |  |
| **Ge06** | | BKPyV |  | no | HSV (CT value 32.0) ** |
| **Ge07** | | JCPyV |  | no | CMV (1.25E1 copies/ml) |
| **Ge08** | | CMV |  | yes (1.60E4 copies/ml) | EBV (1.62E4 copies/ml), CMV (1.62E4 copies/ml) |
|  | | BKPyV |  | no |  |
|  | | JCPyV |  | no |  |
| **Ge09** | | none |  | - | CMV (3.92E1 copies/ml) |
| **Ge10** | | CMV |  | yes (7.91E2 copies/ml) | none |
| **Ge11** | | BKPyV |  | no | none |
| **Ge12** | | BKPyV |  | no | CMV (1.40E2 copies/ml) |
| **Ge13** | | none |  | - | none |
| **Ge14** | | HSV |  | yes (CT value 33.3) ** | none |
|  | | CMV |  | yes (1.59E3 copies/ml) |  |
|  | | HBV |  | yes (5.70E3 copies/ml) |  |
| **Ge15** | | none |  | - | CMV (2.25E1 copies/ml) |
| **Ge16** | | none |  | - | none |
| **Ge17** | | none |  | - | none |
| **Ge18** | | none |  | - | none |
| **Ge19** | | none |  | - | none |
| **Ge20** | | BKPyV |  | no | CMV (9.84E1 copies/ml) |
| **Ge21** | | none |  | - | none |
| **Ge22** | | none |  | - | CMV (2.94E2 copies/ml) |
| **Ge23** | | JCPyV |  | no | CMV (1.23E2 copies/ml) |
| **Ge24** | | none |  | - | EBV (7.22E1 copies/ml) |
| **Ge25** | | BKPyV |  | no | none |

Abbreviations: CT: cycle threshold

^†^ Mean viral loads were calculated based on the results of r(RT-)PCR assays performed as part of routine investigations on the included pooled plasma samples. Of note, results below the lower limit of quantification (LOQ) were considered as negative (LOQ for EBV real-time PCR: <5E2copies/ml; LOQ for CMV real-time PCR : <1.37E2 copies/ml).

* HEV was screened with qualitative RT-PCR only

** HSV was screened with qualitative PCR only

**Figure S1**: Boxplot of mapped reads of DNA viruses and corresponding mRNA detection with mNGS. The vertical axis represents the number of mapped reads. The horizontal axis represents all identified viral sequences of DNA viruses (left panel) and corresponding mRNA sequences (right panel). The numbers on the horizontal axis represent the number of patients in which sequences of each virus were identified.

Abbreviations: HSV-1: herpes simplex 1 virus; CMV: cytomegalovirus; EBV: Epstein-Barr virus; TTMDV: torque teno midivirus; TTMV: torque teno minivirus; TTV: torque teno virus.


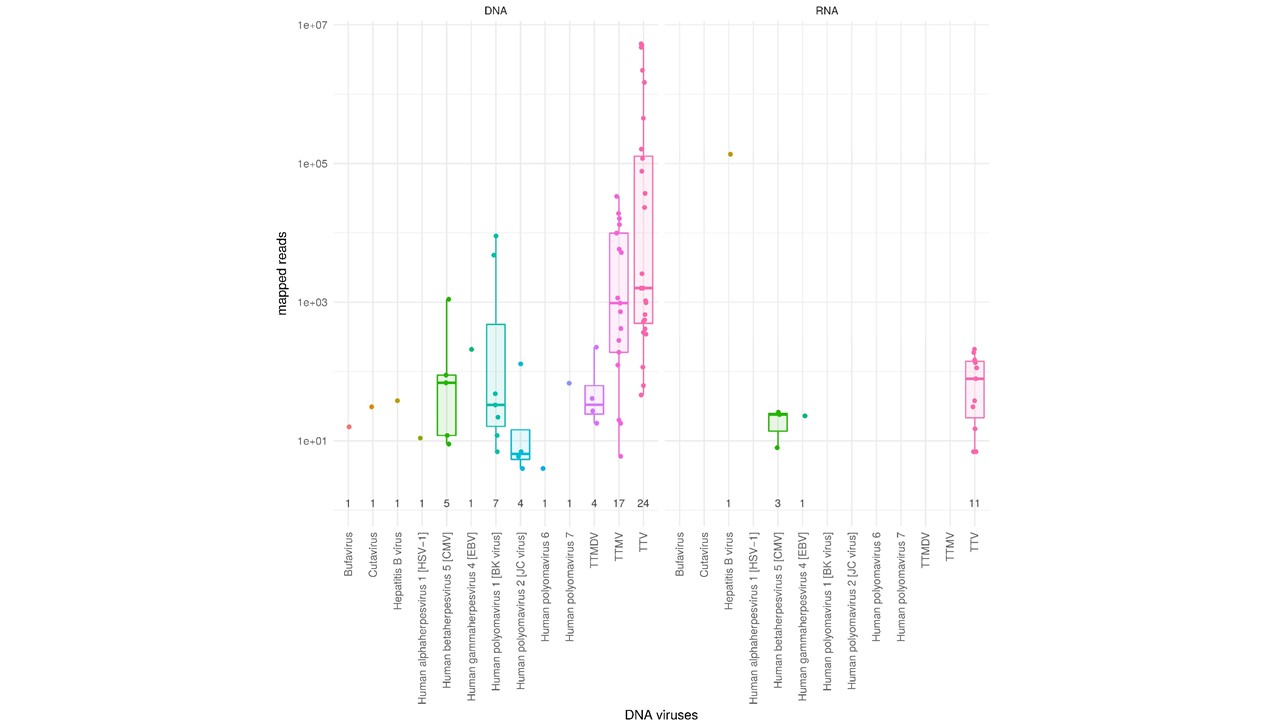

Supplement: Supplementary file 2 — Additional file 1: Table S1. Detailed mNGS results per patient for the 25 adult allo-HSCT patients. Table S2. Comparison of mNGS results on the pooled plasma samples and of the routine r(RT-)PCR results on the corresponding plasma samples of the 25 patients. Figure S1. Boxplot of mapped reads of DNA viruses and corresponding mRNA detection with mNGS. The vertical axis represents the number of mapped reads. The horizontal axis represents all identified viral sequences of DNA viruses (left panel) and corresponding mRNA sequences (right panel). The numbers on the horizontal axis represent the number of patients in which sequences of each virus were identified. Abbreviations: HSV-1: herpes simplex 1 virus; CMV: cytomegalovirus; EBV: Epstein-Barr virus; TTMDV: torque teno midivirus; TTMV: torque teno minivirus; TTV: torque teno virus. [file 40168_2020_953_MOESM2_ESM.docx]
